# Effect of Lignin Removal on the Hygroscopicity of PMMA/Wood Composites

**DOI:** 10.3390/polym14163356

**Published:** 2022-08-17

**Authors:** Fucheng Xu, Linlin Xu, Chaowei Zheng, Yi Wang, Haiyang Zhang

**Affiliations:** 1 College of Materials Science and Engineering, Nanjing Forestry University, Nanjing 210037, China; 2 Jiangsu Co-Innovation Center of Efficient Processing and Utilization of Forest Resources, Nanjing Forestry University, Nanjing 210037, China; 3 International Innovation Center for Forest Chemicals and Materials, Nanjing Forestry University, Nanjing 210037, China

**Keywords:** wood-based composites, delignification, hygroscopicity, BET, H-H model, GDW model

## Abstract

Wood delignification can provide a wood-based template with sufficient pore volume for polymer/wood composites. At the same time, delignification is conducive to the penetration of polymer into the wood cell wall, which is of great significance to improve the function and performance of composites. However, lignin is the main chemical component in wood. The removal of lignin will inevitably lead to the change of the wood’s physical properties, including the hygroscopicity of the wood. In this study, prepolymerized methyl methacrylate (MMA) impregnated delignified wood (DW) was used to obtain polymerized methyl methacrylate/delignified wood (PMMA/DW) composites with different lignin removal. The effect of lignin removal on the hygroscopicity of the composites is discussed. The results of nitrogen adsorption showed that the DW could adsorb more nitrogen than the original wood, and the amount of nitrogen adsorption gradually increased with the improvement of the processing degree. After filling with PMMA, the adsorption amount of nitrogen was greatly reduced. The results of the BET analysis showed that delignification promoted the distribution of PMMA in the pores of the wood cell wall. When lignin was almost completely removed, all mesopores in the cell wall were filled with PMMA. The results of the moisture absorption analysis isotherm curve showed that the moisture absorption content of the wood was positively correlated with the amount of lignin removed, and the moisture absorption content of the PMMA/DW composite was negatively correlated with the amount of lignin removed. The hygroscopic data were further analyzed using the Hailwood–Horrobin model. The results showed that the mole number of adsorbable or hydratable sites of the DW increased with the increase of lignin removal, and the situation of the PMMA/DW composites was just the opposite. In addition, after delignification, the dissolved water content and hydrated water content of the DW increased, and the increase was related to the delignification strength. The increase of dissolved water content indicates that the removal of lignin promotes the production of more volume in the cell wall, which provides space for the adsorption of multilayer water. After MMA in situ polymerization, the hydration and dissolved water content of the PMMA/DW decreased significantly, and the dissolved water content decreased even more significantly. The fitting curves of the H-H model and GDW model for the experimental data points of the differently treated samples were similar. The removal of lignin led to the increase of the w value, indicating that the ratio of water molecules adsorbed in the first layer of DW to the second layer increased, and the removal of lignin resulted in the enhancement of wood hygroscopicity; the opposite was true for the PMMA/DW.

## 1. Introduction

Wood has a natural three-dimensional porous structure, which makes it light and high-strength. It is widely used in transportation, building decoration, and other industries [1,2]. The porous structure of wood also provides a carrier for the filling of functional materials. In recent years, research on polymer/wood composites has been popular [3,4,5]. This kind of material combines the functionality of polymer materials and the natural sustainability of wood and has been widely considered in the fields of high-performance structural materials, energy storage and conversion, environmental remediation, light and heat management, etc. [6,7,8]. However, when preparing polymer/wood composites, the pore structure of the wood itself cannot accommodate more polymer materials. Therefore, researchers usually use the method of delignification to obtain wood with high specific surface area and high porosity [9,10,11,12]. Li et al. [13] obtained optically transparent wood with a transmittance of 85% and a haze of 71% using a delignified nanoporous wood template. Transparent wood was prepared by impregnating the lumen of the cell wall and nanocellulose fiber network with prepolymerized methyl methacrylate (MMA) with refractive index matching. In this process, the hierarchy of wood was preserved. Fu et al. [14] reported a high-performance, multifunctional, and environmentally friendly all-wood flexible electronic circuit film. The substrate adopted a solid, flexible, and transparent wood mold. The manufacturing of the wood mold involved a large amount of removal of lignin and hemicellulose to adjust the nanostructure of the material. This process retained the original arrangement of cellulose nanofibers and promoted their combination. The film was flexible but strong in the fiber direction. The Young’s modulus and tensile strength were 49.9 GPa and 469.9 MPa, respectively. In addition, bio-based inks made of lignin-derived carbon nanofibers were printed on transparent wood films, and the application of strain sensors in printed circuits was demonstrated. The research combined transparent wood film with conductive ink to produce environmentally sustainable wood electronic products for potential applications such as flexible circuits and sensors.

Wood delignification can provide a wood-based template with sufficient pore volume for polymer/wood composites. At the same time, delignification is conducive to the penetration of polymers into wood cell walls to obtain nano-sized functional composites, which is of great significance to the improvement of material properties. However, lignin is the main chemical component in wood. The removal of lignin will inevitably lead to the change of the wood’s physical properties, including the hygroscopicity of the wood. The strength of hygroscopicity also directly affects the dimensional stability of polymer/wood composites. Some scholars have studied the effect of the removal of chemical components in wood on the hygroscopicity of wood. The results showed that the reduction of cellulose and hemicellulose content will enhance the water resistance of the wood, and the reduction of lignin will weaken the water resistance of the wood [15]. Zhou et al. [16] investigated the effect of removing chemical components of lignin and extractive on the equilibrium moisture content of Cunninghamia lanceolata Hook wood and indicated the hygroscopicity of delignification wood was larger than that of the wood with extractive removal. Ou et al. [17] mixed the wood powder treated by extraction, delignification, hemicellulose, and matrix (hemicellulose and lignin) with high-density polyethylene to prepare wood-plastic composites, and investigated its moisture absorption and thickness expansion. The study pointed out that the delignification group had the largest moisture absorption and thickness expansion, followed by the matrix group, extraction group, and hemicellulose removal group.

Previous studies have shown that delignification will enhance the hygroscopicity of wood, which will also have a certain impact on the hygroscopicity of polymer/wood composites. However, there are few studies on this topic, and there are fewer studies on the relationship between the amount of lignin removed and the hygroscopicity of polymer/wood composites. MMA is a common monomer used to make transparent wood based on a delignified template. It has been widely studied because of its refractive index similar to that of cellulose, its low price, and its simple polymerization process [18]. With the increase in lignin removal, the light transmittance of PMMA/wood composites will increase. However, the change in lignin content will also affect the hygroscopicity of the composites. In the present study, PMMA/wood composites with different lignin removal were obtained by impregnating delignified wood with MMA. The effect of lignin removal on the hygroscopicity of the composites is discussed in this paper.

## 2. Materials and Methods

### 2.1. Materials 

Poplar wood (*Populus* L.) was provided by Suqian Hongwei Wood Industry Co., Ltd. (Suqian, China). The wood without tension, biological degradation, discoloration and knots, or other defects was selected as the experimental specimen for testing; its average air-dried density was 0.42 g·cm^−3^ and the average growth ring width was 3.5 mm. Sodium chlorite (NaClO_2_, 80 wt%), glacial acetic acid (CH_3_COOH, AR, 99.5%), sodium acetate (CH_3_COONa, AR, 99%), methyl methacrylate (MMA, CP, 98%), and azobisisobutyronitrile (AIBN, 98%) were purchased from Macleans Biochemical Technology Co., Ltd. (Shanghai, China). The solvents including deionized (DI) water and absolute ethanol were used.

### 2.2. Delignification Process

The poplar (*Populus* L.) sapwood specimens with 10 mm (longitudinal, L) × 10 mm (tangential, T) × 10 mm (radial, R) were selected for the study. Before chemical treatment, all the samples were dried at (103 ± 2) °C for 24 h until reaching a constant mass after being rinsed with DI and ethanol. The dried samples were then extracted with 2 wt% NaClO_2_ and acetate buffer solution (pH 4.6) at 80 °C. The different lignin removal ratio specimens were obtained by adjusting the treatment time to 6, 9, 12, 18, and 24 h. The number of repetitions for individual research variants was five. The delignified liquid was replaced every 6 h to keep the modification efficiency. Subsequently, the extracted samples were soaked in enough distilled water for 12 h to thoroughly remove the residual chemical reagents. After cleaning, the samples were freeze-dried with a vacuum of 20 Pa at −50 °C for 15 h. The specimens were also weighed and recorded as m_1_ (absolute dry weight) after being totally freeze dried. The delignified wood was marked as DW.

### 2.3. Preparation of PMMA/Delignified Wood (PMMA/DW) Composites

A certain amount of MMA monomer was first weighed and 0.3 wt% AIBN was added. The solution was moved into an Erlenmeyer flask and placed in a water bath at 75 °C for 15 min. Then, the flask was quickly cooled with ice water to prevent an explosion. The DW was put into an impregnation tank and kept under 5 kPa for 30 min to extract air from the wood. Subsequently, the MMA prepolymer was sucked into the impregnation tank and submerged samples. Then, the tank was returned to normal atmospheric pressure and kept for 2 h to let the polymer flow into the samples. Next, the impregnated specimens wrapped in the aluminum foil were kept at (75 ± 2) °C for 4 h after removing the excess monomer and then were heated up to (105 ± 2) °C to fully solidify the prepolymer until the weight was constant. The PMMA/delignified wood composite was denoted as PMMA/DW.

### 2.4. Characterization Methods

#### 2.4.1. Determination of Lignin Content

The lignin content of the original wood and DW was determined according to GB/T 747-2003. Firstly, the specimens were extracted by a Soxhlet extractor with a benzene-alcohol mixture (volume ratio 2:1) for 6 h. Secondly, they were placed in a 250 mL Erlenmeyer flask after being air-dried and a pre-prepared (72 ± 0.1)% sulphuric acid solution was added with shocking. The process was maintained for 2 h at 20 °C. Thirdly, all the contents in the 250 mL Erlenmeyer flask were transferred into a 2000 mL Erlenmeyer flask and the acid concentration was diluted to 3% with distilled water. The insoluble matter was precipitated after being boiled for 4 h. After that, the deposits were filtered and washed with hot distilled water until it showed neutral. Finally, the filter paper and residue were moved into a weighing bottle and dried in an oven at (105 ± 3) °C to a constant weight to measure the acid-insoluble lignin. The acid-insoluble lignin content X (%) was calculated according to Equation (1):

(1) $$X\;\left(\%\right)=\frac{m}{m_{1}}\;\times \;100\%$$

where m is the weight of the totally dried acid-insoluble lignin and m_1_ is the weight of the totally dried natural wood and DW.

#### 2.4.2. Nitrogen Adsorption Test

The surface area and pore size distribution were analyzed by a nitrogen adsorption device (ASAP 2020 HD88, Micromeritics, Norcross, GA, USA) at 77 K. Prior to the measurements, samples were out-gassed at 100 °C for 6 h to remove the water vapor. The specific surface area (SBET) was calculated according to the Brunauer–Emmett–Teller method (BET). The pore size distribution was determined by the Barrett–Joyner–Halenda (BJH) method.

#### 2.4.3. Moisture Sorption Test

The moisture sorption was experimentally tested according to ASTME104-85. The specimens were conditioned at 25 °C and six relative humidity (RH) environments controlled by using different saturated salt solutions, including LiCl, MgCl_2_, K_2_CO_3_, NaBr, NaCl, and K_2_SO_4_, corresponding to RH values of 11.3, 32.8, 43.2, 57.6, 75.3, and 97.3%, respectively. The adsorption process started from the 11.3% RH environment and then, after equilibrium for 7 d, moved to the higher RH environment successively. The desorption process started from the 97.3% RH environment and ran in the reverse order. The equilibrium moisture contents (EMCs) were determined based on the weight at each equilibrium state.

#### 2.4.4. Sorption Isotherm Analysis by the Hailwood–Horrobin (H-H) Theory

The adsorption behavior of specimens at each RH was analyzed by fitting the experimental data using the Hailwood–Horrobin (H-H) model [19,20]. The adsorbed water is separated into hydrate water (M_h_) and into dissolved water (M_d_) [21,22]:

(2) $${M=M}_{h}{+M}_{d}=\frac{1800}{W}\left(\frac{K_{h}K_{d}H}{{100+K}_{h}K_{d}H}\right)$$

where M is the percentage of moisture content at a given percentage RH, W is the molecular weight of cell wall polymer per sorption site, K_h_ is the equilibrium constant of the monolayer water formed from dissolved water and cell walls, and K_d_ is the equilibrium constant between water vapor and dissolved water. The values of K_h_ and K_d_ are determined by plotting H/M against H, which is predicted by the H-H theory to give a parabolic relationship of the form shown in Equation (3).

(3) $$\frac{H}{M}=A+\mathrm{BH}\text{-}{\mathrm{CH}}^{2}$$

where K_h_, K_d_, W, A, B, and C are related by the following relationships:

(4) $$A=\frac{W}{18}\left[\frac{1}{K_{d}\left(K_{h}+1\right)}\right]$$

(5) $$B=\left(\frac{W}{1800}\right)\left[\frac{K_{h}\;-\;1}{K_{h}+1}\right]$$

(6) $$C=\left(\frac{W}{180000}\right)\left[\frac{K_{h}K_{d}}{K_{h}+1}\right]$$

#### 2.4.5. Sorption Isotherm Analysis by the Generalized D’Arcy and Watt (GDW) Model

In order to further analyze the hygroscopic behavior of the DW and PMMA/DW, the GDW model was used to analyze the hygroscopic data of samples. The GDW model was also found to be applicable to the description of water sorption data measured on different foodstuffs. The GDW can be written in the following form [23,24]:

(7) $$M=\frac{mKh_{r}}{1+Kh_{r}}\cdot \frac{1-k\left(1-w\right)h_{r}}{1-kh_{r}}$$

where *M* is the moisture content in wood, *h_r_* is the relative humidity, *m* is the concentration of primary active surface sites, and *K* and *k* are the kinetic constants related to the sorption on primary and secondary sorption sites, respectively. The coefficient *w* determines the ratio of water molecules sorbed on the primary sites which is converted into the secondary sorption sites. The evaluation indicators of the fitting effect of the model include the determination coefficient R^2^ and root mean square RMSE. When the R-square value is the largest and the RMSE value is the smallest, the fitting effect is considered to be the best.

(8) $$R^{2}=1-\frac{\sum_{i}^{n}{\left(M_{i}^{o}-M_{i}^{t}\right)}^{2}}{\sum_{i}^{n}{\left(M_{i}^{o}-\bar{M^{o}}\right)}^{2}}$$

(9) $$RMSE=\sqrt{\frac{\sum_{i}^{n}{\left(M_{i}^{o}-M_{i}^{t}\right)}^{2}}{n-1}}\;$$

where $M_{i}^{o}$ is the observed moisture content for the ith experimental point, $M_{i}^{t}$ is the theoretical value of the moisture content calculated from Equation (7), $\bar{M^{o}}$ is the average observed moisture content, and n is the number of data points.

## 3. Results and Discussion

### 3.1. Lignin Removal Process

Table 1 shows the lignin content changes of the modified wood and the corresponding lignin removal level. More lignin in the wood cell wall was removed as the modification time was prolonged.

### 3.2. Pore Characteristics

**Figure 1:**
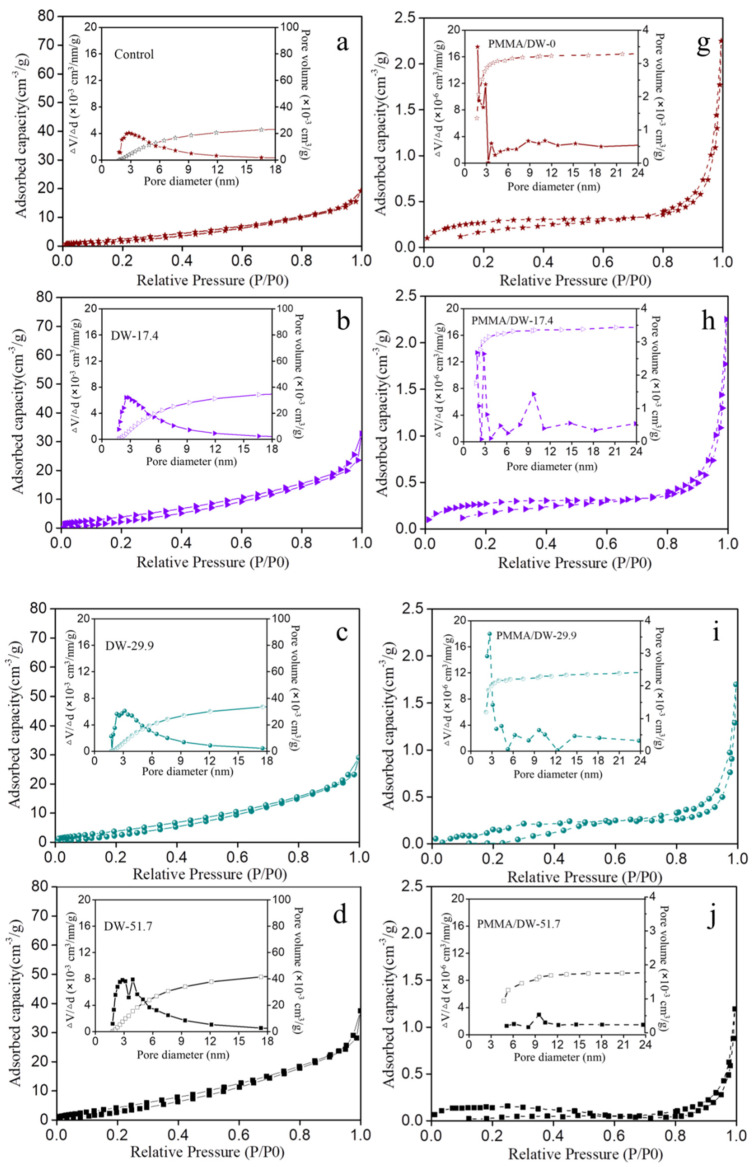

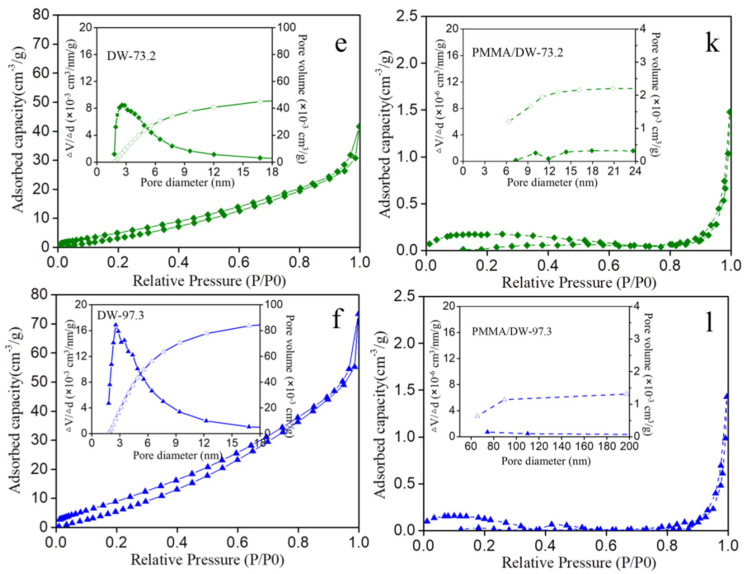
Nitrogen adsorption–desorption isotherms and mesopore-size distribution and accumulated pore volume: (**a**–**f**) DW and (**g**–**l**) PMMA/DW.

In order to obtain information on the pore structure of the wood and PMMA/DW composites with different amounts of lignin removal, the nitrogen adsorption method was used for analysis in this study. The nitrogen adsorption and desorption isotherms of the control wood, DW, and PMMA/DW are shown in Figure 1. Obviously, the DW could absorb more nitrogen and the nitrogen adsorption capacity gradually increased with the improvement of processing degree. This is similar to the results of previous studies [15]. After being impregnated with MMA and solidified, the adsorption of nitrogen was greatly reduced. In addition, hysteresis was very obvious in all the DW samples while in the polymer impregnated DW was very limited. Figure 1a–f shows the pore size distribution in the range of 2–18 nm of the natural wood and DW and the accumulative pore volume in the wood cell wall. The peak appeared at about 3 nm in the original wood, indicating that there were more mesopores in this diameter range, which was similar to the results of previous research [25,26]. The number of mesopores around 3 nm increased with delignification, as shown by the increased intensity of the peak there. Furthermore, a new peak appeared near 4 nm, indicating that delignification treatment widened the diameter of the original smaller mesopores. The comparison of the DW and PMMA/DW in Figure 1 shows the changes in pore size and volume after adding PMMA. Compared with PMMA/DW-0, PMMA/DW-17.4 had an obvious peak at 10 nm and PMMA/DW-29.9 had a weaker peak at 10 nm. Furthermore, when the lignin removal level was lower than 29.9%, the peak at 3 nm still remained, suggesting that the PMMA distribution efficiency in smaller 3 nm mesopores was much lower than in the larger 10 nm mesopores. As lignin removal increased, the 3 nm peak almost disappeared.

The above results revealed that when the lignin removal was lower than 29.9%, the PMMA distribution efficiency in smaller mesopores was much lower than that in larger mesopores. For the pore sizes of 3 nm and 10 nm, respectively, when the lignin removal exceeded 29.9%, the PMMA distribution efficiency in the two mesopores showed the opposite pattern. After PMMA occupied the cell wall, the number of mesopores in the DW within a 29.9–51.7% lignin removal was reduced. When lignin removal reached 97.3%, all the mesopores detected in this study disappeared. In combination with Table 2, it can be seen that delignification promoted the distribution of PMMA in the wood cell wall pores. When the lignin was almost completely cleared away, all the mesopores in the cell walls were charged with PMMA. The natural pores enlarged to the diameter of 3–4 nm mixed in the cellulose microfibrils due to the removal of lignin were unveiled to the non-polar monomers. After being impregnated with MMA, not only the commonly known cell cavities, but also the cell wall pores, were refilled.

### 3.3. Comparison of the Sorption Isotherm of the DW and PMMA/DW

**Figure 2:**
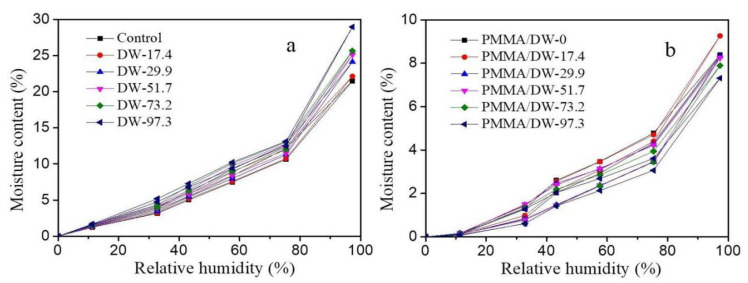
Moisture content change with relative humidity of DW (**a**) and PMMA/DW (**b**) during adsorption and desorption process.

Moisture content (MC) change with the relative humidity of the DW and PMMA/DW is presented in Figure 2. All curves showed similar sigmoid shapes. Obviously, MC was higher for the DW than the control wood and, as the lignin removal increased, the MC changed more, indicating delignification can increase the MC changes. Lignin is relatively hydrophobic due to its molecular structure and distribution. However, as mentioned above, more mesopores are produced in the wood after delignification. The increase in SBET in Table 2 confirmed this, and previous studies have also confirmed that more hydroxyl groups can be obtained in the wood after delignification [9]. After PMMA filling, the MC of the composites was obviously reduced (Figure 2b). This is due to the in situ polymerization of MMA resin in the wood cell wall, and PMMA forms a coating on the adsorption sites of cellulose and hemicellulose, which reduces the SBET and pore volume (Table 2), thereby reducing the adsorption of moisture.

### 3.4. Moisture Sorption Analysis Using the Hailwood–Horrobin Model

The moisture sorption data were further analyzed using Hailwood–Horrobin model. The fitted and physical constants calculated for the Hailwood–Horrobin adsorption isotherms of the DW and PMMA/DW are listed in Table 3. The R^2^ values ranged from 0.860 to 0.982, indicating a good fit to the experimental results. In past research, the fitting results of wood hygroscopicity also had a good performance [27]. The 1/W was also calculated since it represents the number of moles of adsorption or hydration sites per gram of wood or composite. For the DW, the 1/W values increased as the lignin removal enhanced, indicating that a proportion of sites were made available for water sorption. The opposite was true for the PMMA/DW. When the lignin removal was from 0 to 17.4%, the 1/W of PMMA/DW increased, and when the lignin removal reached 29.9%, the 1/W value of PMMA/DW-29.9 started to decrease, which indicated that a proportion of sites were made unavailable for water sorption. In addition, comparing the control wood with the PMMA/DW-97.3 composite, the 1/W of PMMA/DW-97.3 was much lower than that of the control wood, which indicated that the hygroscopicity of the composite was greatly reduced after the complete exchange of lignin and PMMA.

**Figure 3:**
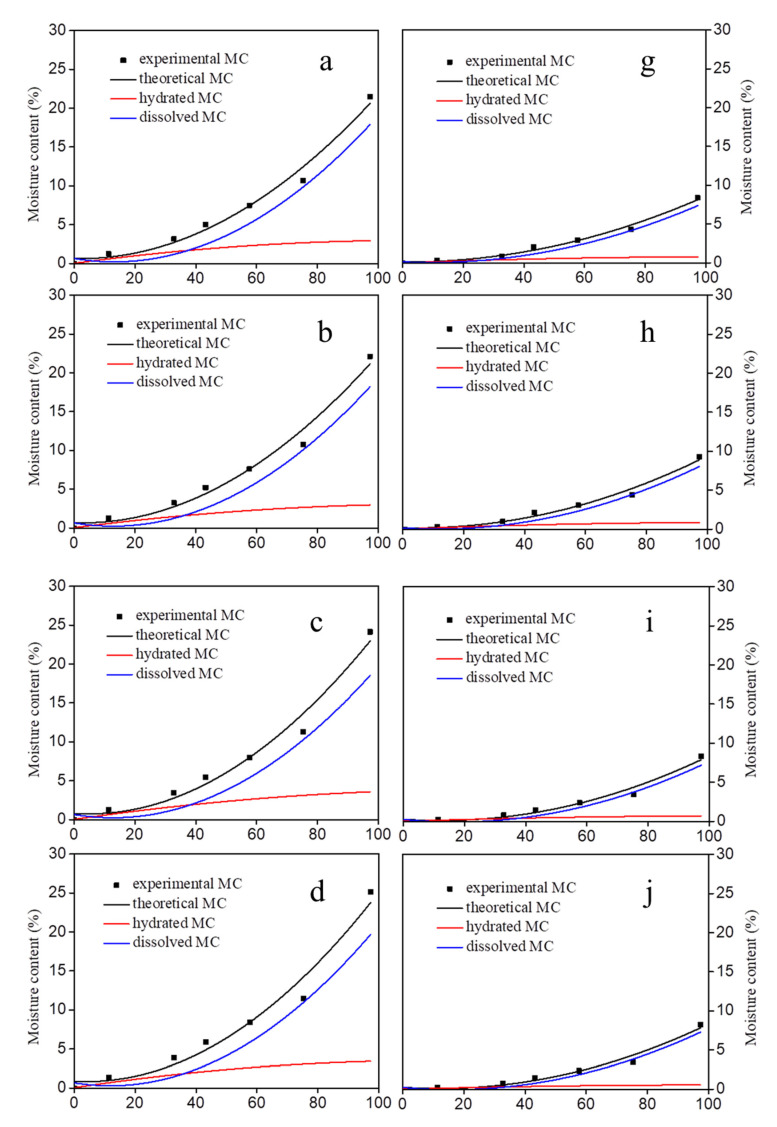

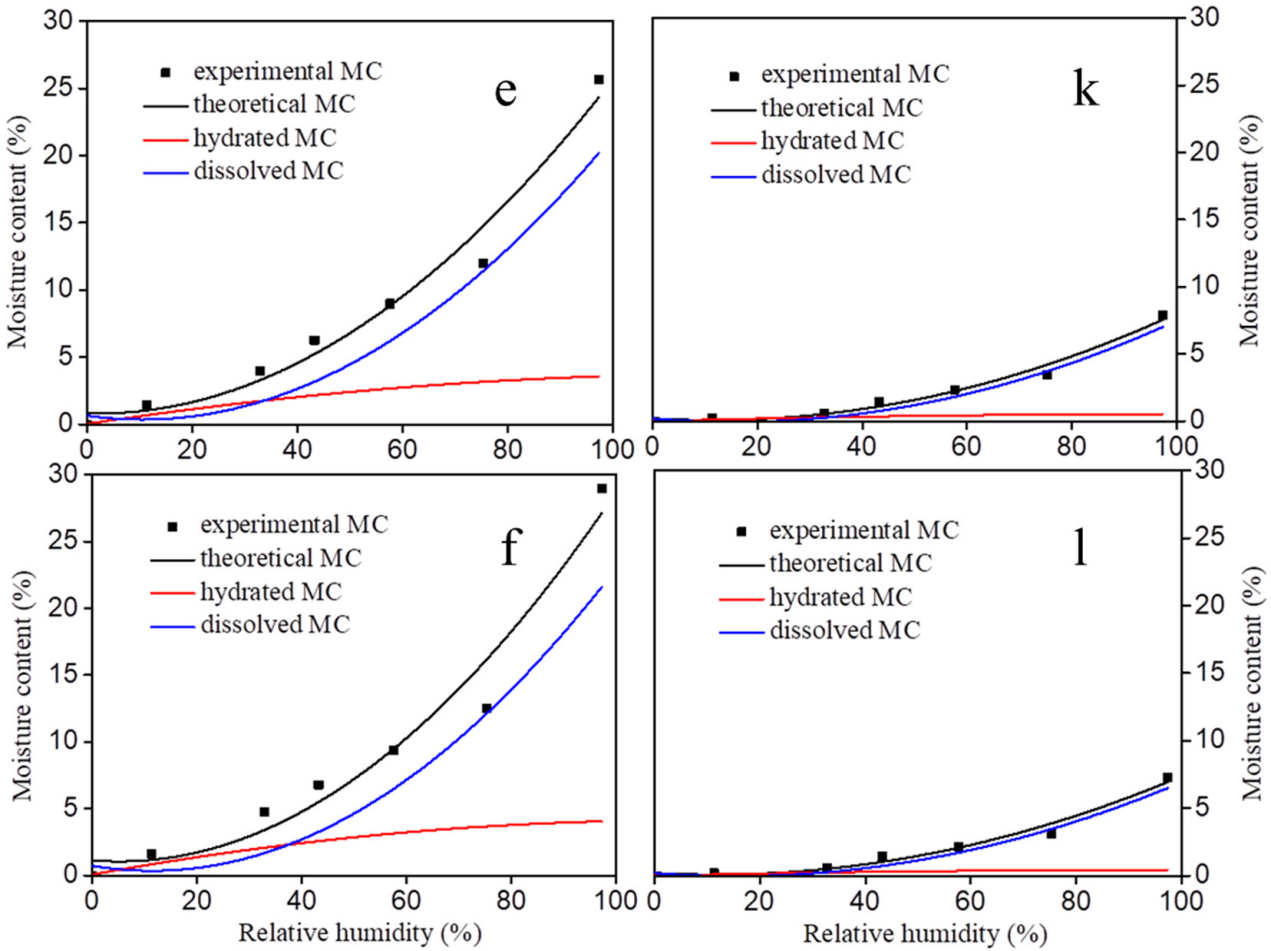
Hydrated, dissolved moisture content (MC) calculated with the Hailwood–Horrobin model and the sum of hydrated and dissolved (theoretical) adsorption isotherms through the relative humidity run compared to the experimental MC of DW (**a**–**f**) and PMMA/DW (**g**–**l**).

The fitted curve using the H-H model coincided well with the experimental data adsorption isotherm of the DW and PMMA/DW (Figure 3). Through the use of the H-H model, the adsorbed water was separated into hydrate water (M h) and into dissolved water (M d). After delignification, the hydrated MC and dissolved MC of the DW gradually increased (Figure 3a–f), and the increase was related to the intensity of delignification. The increase of hydrated MC and dissolved MC indicated that with the removal of lignin, more hydroxyl groups were produced in the cell wall [9]. The increase in dissolved MC indicated that the removal of lignin promoted the generation of more volume in the cell wall, which provided space for the adsorption of multilayer water. This result was consistent with the increase in the total pore volume of the DW in Table 2. After the in situ polymerization of MMA, the hydrated MC and dissolved MC of the PMMA/DW were obviously reduced and the decrease of dissolved MC was more obvious (Figure 3g–l). The reduction in dissolved MC presumably reflected the decreased capacity of expansion of the cell wall owing to increased matrix stiffness and increased PMMA cross-linking.

### 3.5. Moisture Sorption Analysis Using the GDW Model

**Figure 4:**
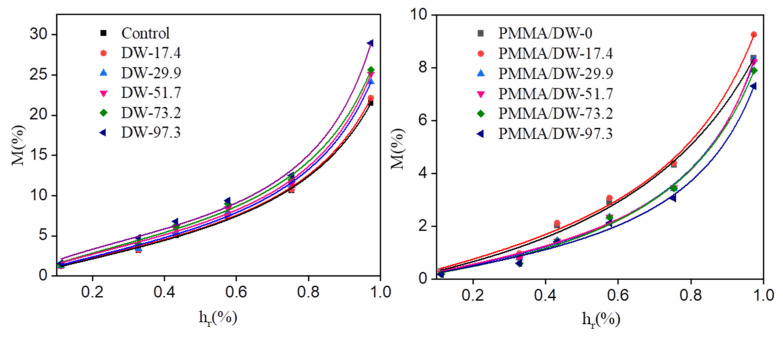
The results of the fitting of studied experimental data by the GDW model.

Figure 4 show the graphical representation of the fitting of the studied experimental data by the GDW model, and Table 4 collects the values of the best fit parameters. The fit of the GDW model to the experimental data was satisfactory (R-square was equal to 0.99 or more). The fitting curves of the H-H model and GDW model for the experimental data points of the different treated samples were similar. The value of the kinetic constant K of the first layer of water adsorption exceeded 1, indicating that the wood was relatively sensitive to humidity. The value of the second layer water adsorption kinetic constant k was less than 1, and the value of the PMMA/DW k was less than that of the DW, indicating that the sensitivity of the PMMA/DW to the second layer water adsorption was lower than that of the DW. The W value of the DW is directly related to the amount of lignin removed. The removal of lignin led to the increase of the w value, indicating that the ratio of water molecules adsorbed in the first layer of the DW to the second layer increased, and the removal of lignin resulted in the enhancement of wood hygroscopicity. The w value of the PMMA/DW decreased, indicating that the amount of water molecules converted from the first layer of the PMMA/DW to the second layer was less, and the hygroscopicity of the PMMA/DW gradually decreased. By using the GDW model to analyze the water adsorption data of the sample, the results further confirm the conclusion of the H-H model.

## 4. Conclusions

In this paper, MMA impregnated delignified wood (DW) was used to obtain PMMA/delignified wood (PMMA/DW) composites with different lignin removal. The effect of lignin removal on the hygroscopicity of the composites was discussed. The results of nitrogen adsorption showed that DW could adsorb more nitrogen than the original wood, and the amount of nitrogen adsorption gradually increased with the improvement of processing degree. After filling with PMMA, the adsorption amount of nitrogen was greatly reduced. The results of BET analysis showed that delignification promoted the distribution of PMMA in the pores of the wood cell wall. When lignin was almost completely removed, all mesopores in the cell wall were filled with PMMA. The results of the moisture absorption analysis isotherm curve showed that the moisture absorption content of wood was positively correlated with the amount of lignin removed, and the moisture absorption content of the PMMA/DW composite was negatively correlated with the amount of lignin removed. The hygroscopic data were further analyzed using the Hailwood–Horrobin model. The results showed that the mole number of adsorbable or hydratable sites of DW increased with the increase of lignin removal, and the situation of PMMA/DW composites was just the opposite. In addition, after delignification, the dissolved water content and hydrated water content of DW increased, and the increase was related to the delignification strength. The increase of dissolved water content indicates that the removal of lignin promotes the production of more volume in the cell wall, which provides space for the adsorption of multilayer water. After MMA in situ polymerization, the hydration and dissolved water content of PMMA/DW decreased significantly, and the dissolved water content decreased even more significantly. The fitting curves of the H-H model and GDW model for the experimental data points of the different treated samples were similar. The removal of lignin led to the increase of the w value, indicating that the ratio of water molecules adsorbed in the first layer of DW to the second layer increased, and the removal of lignin resulted in the enhancement of wood hygroscopicity, while the opposite was true for PMMA/DW.

## Figures and Tables

**Table 1:** The relationship between lignin removal and treatment time.

Time (h)	Lignin Content (wt%)	Lignin Removal (%)
0	22.8 (0.9)	0
6	18.83 (1.27)	17.4
9	15.98 (2.2)	29.9
12	11.01 (1.76)	51.7
18	6.11 (0.22)	73.2
24	0.62 (0.03)	97.3

**Table 2:** Specific surface area (SBET) and total pore volume (V total) of the delignified wood (DW) and polymethyl methacrylate/delignified wood (PMMA/DW).

Simples	SBET (m^2^/g)	V Total (×10^−3^ cm^−3^/g)
Control	15.1298	27.1
DW-17.4	23.6308	40.1
DW-29.9	27.0282	41.2
DW-51.7	31.8868	47.8
DW-73.2	36.2904	54.3
DW-97.3	71.3155	94.8
PMMA/DW-0	0.9975	3.482
PMMA/DW-17.4	1.1676	3.560
PMMA/DW-29.9	1.4671	2.623
PMMA/DW-51.7	0.5046	1.848
PMMA/DW-73.2	0.5848	2.288
PMMA/DW-97.3	0.2946	1.526

**Table 3:** Fitted and physical constants calculated for the Hailwood–Horrobin adsorption isotherms of the delignified wood (DW) and polymethyl methacrylate/delignified wood (PMMA/DW).

Simples	A	B	C	K_h_	K_d_	W (g/mol)	1/W (mmol/g)	R^2^
Control	9.320	0.0263	0.000780	1.360	0.785	310.572	3.220	0.968
DW-17.4	9.230	0.0216	0.000730	1.300	0.780	298.052	3.355	0.973
DW-29.9	8.728	0.0242	0.000740	1.350	0.793	292.579	3.418	0.886
DW-51.7	8.042	0.0218	0.000645	1.352	0.770	262.230	3.813	0.912
DW-73.2	7.841	0.0136	0.000550	1.230	0.755	237.676	4.207	0.961
DW-97.3	6.717	0.0266	0.000597	1.517	0.765	232.944	4.293	0.860
PMMA/DW-0	45.362	0.500	0.00154	5.392	0.251	1309.59	0.764	0.894
PMMA/DW-17.4	41.383	0.451	0.00140	5.323	0.252	1187.38	0.842	0.882
PMMA/DW-29.9	53.853	0.559	0.00136	6.103	0.203	1400.58	0.714	0.982
PMMA/DW-51.7	59.937	0.728	0.00247	5.395	0.276	1906.78	0.524	0.956
PMMA/DW-73.2	65.539	0.765	0.00219	5.908	0.238	1938.11	0.516	0.968
PMMA/DW-97.3	71.355	0.926	0.00345	5.294	0.302	2443.08	0.409	0.961

**Table 4:** The values of the best fit parameters obtained from the fitting of experimental data by the GDW model.

Simples	m (%)	K	k	w	R^2^
Control	3.410	2.462	0.791	0.130	0.9966
DW-17.4	3.573	2.550	0.812	0.155	0.9957
DW-29.9	3.423	2.372	0.823	0.207	0.9943
DW-51.7	3.964	2.834	0.859	0.221	0.9946
DW-73.2	4.359	2.611	0.887	0.253	0.9967
DW-97.3	4.621	2.987	0.904	0.271	0.9921
PMMA/DW-0	0.641	5.813	0.349	0.009	0.9907
PMMA/DW-17.4	0.762	5.834	0.338	0.012	0.9896
PMMA/DW-29.9	0.696	5.973	0.197	0.008	0.9920
PMMA/DW-51.7	0.482	5.814	0.284	0.006	0.9953
PMMA/DW-73.2	0.491	5.781	0.212	0.004	0.9911
PMMA/DW-97.3	0.380	5.427	0.291	0.003	0.9954

## Data Availability

The data presented in this study are available upon request from the corresponding author.
